# Development of a New Positron Emission Tomography Imaging Radioligand Targeting RIPK1 in the Brain and Characterization in Alzheimer's Disease

**DOI:** 10.1002/advs.202309021

**Published:** 2024-06-25

**Authors:** Ping Bai, Yu Lan, Yan Liu, Prasenjit Mondal, Ashley Gomm, Yulong Xu, Yanli Wang, Yongle Wang, Leyi Kang, Lili Pan, Frederick A. Bagdasarian, Madelyn Hallisey, Fleur Lobo, Breanna Varela, Se Hoon Choi, Stephen N. Gomperts, Hsiao‐Ying Wey, Shiqian Shen, Rudolph E. Tanzi, Changning Wang, Can Zhang

**Affiliations:** ^1^ Department of Pulmonary and Critical Care Medicine Targeted Tracer Research and Development Laboratory Institute of Respiratory Health Frontiers Science Center for Disease‐related Molecular Network Precision Medicine Key Laboratory of Sichuan Province & Precision Medicine Research Center West China Hospital Sichuan University Chengdu Sichuan 610041 China; ^2^ Athinoula A. Martinos Center for Biomedical Imaging Department of Radiology Massachusetts General Hospital Harvard Medical School Charlestown MA 02129 USA; ^3^ Department of Pharmacy Renmin Hospital of Wuhan University Wuhan 430060 China; ^4^ Genetics and Aging Research Unit McCance Center for Brain Health MassGeneral Institute for Neurodegenerative Disease Department of Neurology Massachusetts General Hospital Harvard Medical School 114 16th Street Charlestown MA 02129 USA; ^5^ Department of Nuclear Medicine Laboratory of Clinical Nuclear Medicine West China Hospital Sichuan University Chengdu 610041 China; ^6^ Department of Neurology Massachusetts General Hospital Harvard Medical School 114 16th Street Charlestown MA 02129 USA; ^7^ Department of Anesthesia Critical Care and Pain Medicine Massachusetts General Hospital Harvard Medical School Charlestown MA 02129 USA

**Keywords:** [^11^C]CNY‐10, Alzheimer's disease, Necroptosis, Neuroinflammation, Positron emission tomography, Radioligand, RIPK1

## Abstract

Targeting receptor‐interacting protein kinase 1 (RIPK1) has emerged as a promising therapeutic stratagem for neurodegenerative disorders, particularly Alzheimer's disease (AD). A positron emission tomography (PET) probe enabling brain RIPK1 imaging can provide a powerful tool to unveil the neuropathology associated with RIPK1. Herein, the development of a new PET radioligand, [^11^C]CNY‐10 is reported, which may enable brain RIPK1 imaging. [^11^C]CNY‐10 is radiosynthesized with a high radiochemical yield (41.8%) and molar activity (305 GBq/µmol). [^11^C]CNY‐10 is characterized by PET imaging in rodents and a non‐human primate, demonstrating good brain penetration, binding specificity, and a suitable clearance kinetic profile. It is performed autoradiography of [^11^C]CNY‐10 in human AD and healthy control postmortem brain tissues, which shows strong radiosignal in AD brains higher than healthy controls. Subsequently, it is conducted further characterization of RIPK1 in AD using [^11^C]CNY‐10‐based PET studies in combination with immunohistochemistry leveraging the 5xFAD mouse model. It is found that AD mice revealed RIPK1 brain signal significantly higher than WT control mice and that RIPK1 is closely related to amyloid plaques in the brain. The studies enable further translational studies of [^11^C]CNY‐10 for AD and potentially other RIPK1‐related human studies.

## Graphical abstract

This study presents a newly developed positron emission tomography (PET) radioligand, named [^11^C]CNY‐10, specifically targeting brain RIPK1. [^11^C]CNY‐10 exhibits favorable properties in preclinical evaluations, laying the groundwork for further clinical translation studies. Moreover, the findings demonstrate that [^11^C]CNY‐10 can be used for research on the diagnosis and pathological mechanisms of Alzheimer's disease (AD) and potentially other RIPK1‐related diseases.

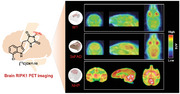

## 1 Introduction

**Figure 1:**
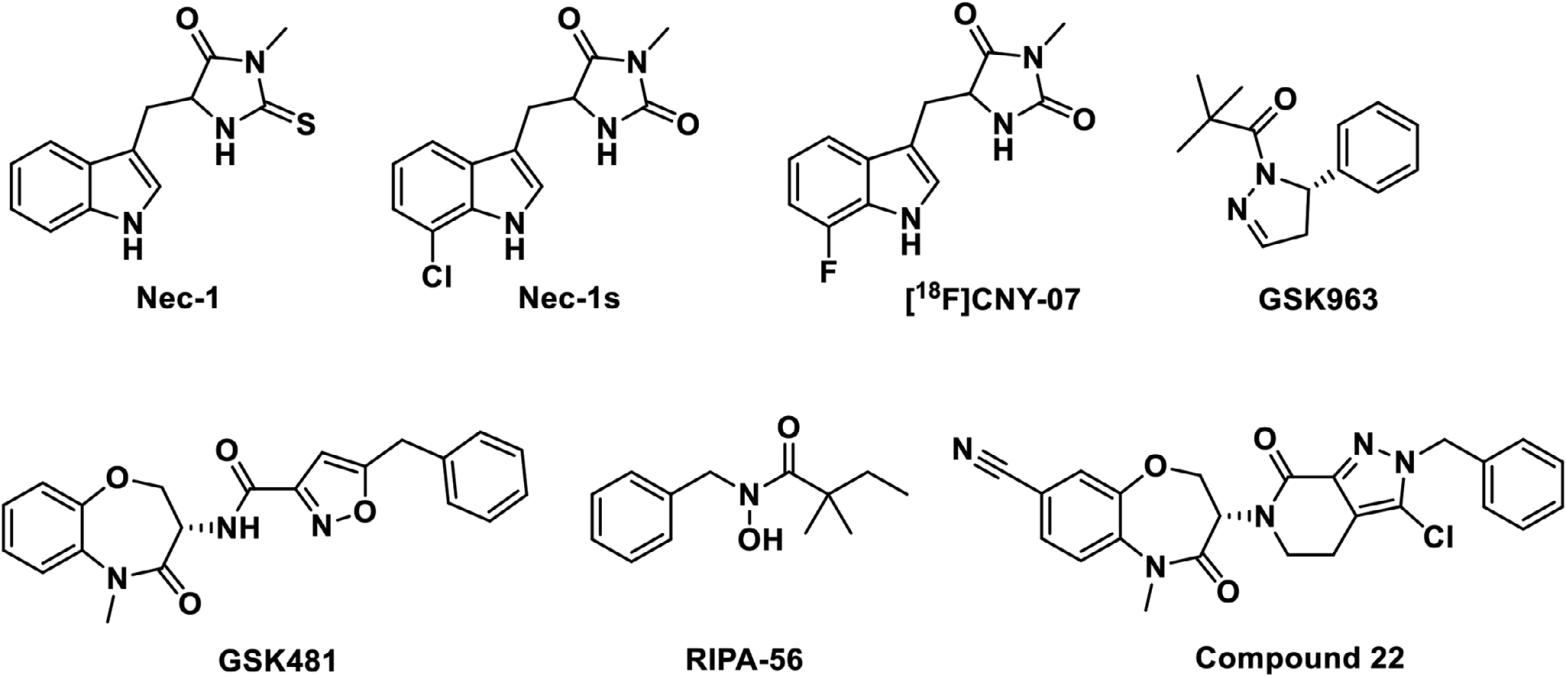
Representative RIPK1 inhibitors and radioligand [^18^F]CNY‐07.

Apoptosis is a form of caspase‐dependent non‐inflammatory programmed cell death that is strictly regulated by intracellular signaling molecules^[^ ^1^ ^]^ Unlike apoptosis, necrosis is a type of non‐programmed cell death that is a passive death mode that occurs under uncontrollable extreme external conditions.^[^ ^2^, ^3^ ^]^ These two forms of cell death have been regarded as the primary forms of cell death in recent decades. With the ongoing study of the underlying mechanism of apoptosis, an increasing number of studies reported that necrosis is controllable under certain circumstances. In 2005, Yuan et al. found that the small molecule necrostatin 1 (Nec‐1, **Figure** **1**) could specifically inhibit cell necrosis without affecting apoptosis mediated by factor‐associated suicide (Fas) and tumor necrosis factor receptor 1 (TNFR1)^[^ ^4^ ^]^ This finding revealed that necrosis could be controlled, and this subset of programmed cell death was named necroptosis.

Necroptosis is a type of caspase‐dependent cell death that can activate the immune response and cause inflammation.^[^ ^5^, ^6^ ^]^ The upstream receptor‐interacting protein kinase 1 (RIPK1) plays a crucial role in regulating cell survival, death, and inflammatory activation in necroptosis^[^ ^7^ ^]^ In RIPK1‐dependent necroptosis signaling, transforming growth factor‐β‐activated kinase 1 (TAK1) and TAK1‐binding proteins (TABs) are recruited by ubiquitinated RIPK1 to form the TAK1‐TAB2‐TAB3 complex, activating the NF‐κB pathway to produce cytokines and inhibit cell death.^[^ ^8^, ^9^ ^]^ On the other hand, when RIPK1 is deubiquitinated, it can form a complex with Fas‐associated proteins via a death domain (FADD) and precursor caspase‐8, which activates cell apoptosis.^[^ ^9^, ^10^ ^]^ The inhibition of caspase 8 (such as ubiquitin inhibitor zVAD) can activate RIPK3, forming a complex containing RIPK1, RIPK3, FADD, precursor caspase‐8, and a mixed lineage kinase domain‐like protein (MLKL).^[^ ^11^, ^12^ ^]^ Phosphorylation of this complex triggers the disruption of cell membrane permeability and initiates necroptosis. As a key regulator in the necroptosis signaling pathway, RIPK1 is involved in the regulation of inflammatory cytokines and has been shown to be associated with a variety of human diseases, including immune diseases, cancer, and neurodegenerative diseases.^[^ ^13^, ^14^, ^15^ ^]^

Inhibition of RIPK1 has exhibited promising therapeutic potential in many diseases. Since the first RIPK1 inhibitor Nec‐1 was discovered, a number of RIPK1 inhibitors have been reported for the treatment of different diseases, especially neurological disorders.^[^ ^4^, ^16^, ^17^, ^18^, ^19^, ^20^ ^]^ For example, RIPK1 was reported to be a promising therapeutic target for Alzheimer's disease (AD) treatment.^[^ ^21^, ^22^ ^]^ AD is the most common neurodegenerative disorder without effective treatment. Although the pathological mechanism of AD remains unclear, multiple factors, such as genetics, aging, and neuroinflammation, contribute to the occurrence and development of AD.^[^ ^23^, ^24^ ^]^ The identification of RIPK1 as a key mediator of both necroptosis and microglial inflammation in AD provides a new strategy for disease diagnosis and treatment. Recent studies found that RIPK1 is upregulated by microglial cells in human AD brains, and pharmacological inhibition of RIPK1 resulted in reduced amyloid pathology, normalized inflammatory changes, and significantly rescued memory deficits in the APP/PS1 transgenic mouse model^[^ ^22^ ^]^ Further studies suggested that RIPK1 is up‐regulated in human AD brains and mediates a disease‐associated microglial response (DAM) in AD, which is related to an inflammatory response and a reduction in phagocytic activity.^[^ ^21^, ^25^ ^]^ As a result, RIPK1 has emerged as an important biomarker and therapeutic target for both etiology understanding and pharmaceutical treatment of AD.

Positron emission tomography (PET) is a powerful molecular imaging tool that has been widely used in both preclinic and clinic for disease diagnosis, target investigation, and pharmacological performance evaluation in drug treatment.^[^ ^26^, ^27^ ^]^ Multiple PET probes for amyloid and tau pathology have been reported and routinely used in drug development and clinical evaluation of AD. Since neuroinflammation is considered to be a critical process associated with AD and various neurogenerative disorders, there has been keen interest in the field to develop PET probes for neuroinflammation; unfortunately, almost all clinically reported PET probes lack promising results in patients largely due to complexity of immune responses in the brain and the need of specific molecular probes.^[^ ^28^, ^29^ ^]^ Given the close relationship between RIPK1 and neuroinflammation in AD, PET imaging of RIPK1 in the brain could provide a practical way to investigate the pathophysiological role of RIPK1, which will be helpful for the diagnosis, treatment, and pathological analysis of AD. In general, although PET imaging has certain limitations, such as high cost, limited availability, and radiation exposure, it is considered one of the most practical, reliable, and widely used non‐invasive methods for preclinical and clinical studies of AD. However, there are no RIPK1 PET probes that can be used in the clinic at present. Toward this unmet need, we have been focused on developing a series of promising RIPK1 probes and recently reported the first RIPK1 fluorine‐18 labeled PET radiotracer ([^18^F]CNY‐07), which showed good brain penetration and binding specificity in rodents^[^ ^30^ ^]^ In this work, we describe the development and preclinical evaluation of a carbon‐11 (^11^C) labeled Nec‐1s (Figure 1) PET radiotracer targeting RIPK1, named [^11^C]CNY‐10. It displayed excellent brain uptake, binding specificity and selectivity, and appropriate kinetics in rodents. Moreover, [^11^C]CNY‐10 showed high brain uptake and heterogeneous distribution in the brain of non‐human primates (NHP). Importantly, [^11^C]CNY‐10 also displayed higher radioactivity uptake in AD transgenic mice relative to the wild‐type (WT) mice, consistent with reported ex vivo postmortem results. Our studies demonstrate that [^11^C]CNY‐10 is a promising PET radioligand for RIPK1 imaging in AD and potentially in other neurological disorders.

## 2 Results and Discussion

### 2.1 Pharmacological and Physicochemical Properties of Nec‐1s

Nec‐1s is a selective RIPK1 inhibitor bearing a tryptophan‐like scaffold. It has been extensively investigated in the RIPK1‐related disease models in preclinic.^[^ ^31^, ^32^ ^]^ Previous studies have demonstrated that Nec‐1s possesses excellent binding affinity and selectivity against RIPK1 with a *K*_d_ value of 3.1 nm and no significant binding for other 485 human kinases^[^ ^22^ ^]^ Furthermore, Nec‐1s exhibited brain‐blood barrier (BBB) permeability with a brain‐to‐plasma ratio (B/P) of 2.4 at 30 min post‐injection in mice^[^ ^33^ ^]^ Additionally, we examined the physicochemical properties of Nec‐1s, including molecular weight, total polar surface area (tPSA), and lipophilicity (**Table** **1**), to predict its in vivo performance as a PET ligand. As a result, Nec‐1s exhibited favorable physicochemical parameters for BBB penetration and binding specificity. The pharmacological and physicochemical properties of Nec‐1s indicate its great potential as a promising PET radioligand for brain RIPK1 imaging.

**Table 1:** The pharmacological and physicochemical properties of Nec‐1s^a^ ^)^.

Molecular weight	tPSA	cLog P	Log *D* [pH = 7.4]	RIPK1 binding affinity (*K*_d_, nm)	Binding selectivity
277.7	61.4	1.7	2.1	3.1	>1000‐fold

^a)^ Molecular weight, tPSA, and cLogP value of Nec‐1s were calculated using ChemBioDraw 20.0; Log *D* value was quantified in n‐octanol/phosphate buffer (pH 7.4) by the shake‐flask method.

### 2.2 Synthesis of Precursor and Preparation of [^11^C]CNY‐10

**Scheme 1:**
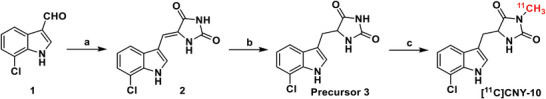
The synthesis of precursor and preparation of [^11^C]CNY‐10. Reagents and conditions: a) imidazolidine‐2,4‐dione, piperidine, 110 °C, 6 h; b) NaBH_4_, THF, 0 °C – rt, 12 h; c) **3** (precursor, 0.5 mg), [^11^C]CH_3_I, K_2_CO_3_ (5.0 mg), in 0.3 mL DMF, 3 min, 60 °C. Radiochemical yield (RCY): 33.4–44.2% (*n* = 4, non‐decay corrected to trapped [^11^C]CH_3_I).

Given that there is one N‐methyl group on the imidazolidinedione of Nec‐1s, we employed N‐methylation of its dimethyl precursor **3** reacting with [^11^C]methyl iodide to prepare [^11^C]CNY‐10 (**Scheme** **1**). To synthesize precursor **3**, the commercially available starting material 7‐chloro‐1H‐indole‐3‐carbaldehyde (**1**) was reacted with imidazolidine‐2,4‐dione in the presence of piperidine to form the intermediate (**2**). Precursor **3** was prepared by the reduction of intermediate using NaBH_4_.

Because of three methylation reaction sites presenting on precursor **3**, the radiochemical yield (RCY) of [^11^C]CNY‐10 differed remarkably under different bases and temperatures. As such, we screened the radiolabeling conditions by changing bases and reaction temperatures. As shown in **Table** **2**, harsh conditions within a strong base and high temperature resulted in a low RCY of [^11^C]CNY‐10. In contrast, a relatively weak base and low temperature remarkably improved the RCY of [^11^C]CNY‐10. Finally, we radiosynthesized [^11^C]CNY‐10 by treating precursor **3** with [^11^C]CH_3_I in the presence of K_2_CO_3_ at 60 °C for 3 min, with an average RCY of 41.8% (non‐decay corrected to trapped [^11^C]CH_3_I). The crude product was purified by semipreparative high‐performance liquid chromatography (semi‐HPLC). [^11^C]CNY‐10 was prepared in 35–40 min at the end of bombardment (EOB) with good high radiochemical purity (>95%) and molar activity (305 GBq µmol^−1^).

**Table 2:** The radiochemical yield of [^11^C]CNY‐10 under different conditions^a^ ^)^.

Bases	Time [min]	Temp [°C]	Solvent [0.3 mL]	RCY [%]
K_2_CO_3_ (5 mg)	3	100	DMF	12.5 ± 3.6
NaOH (5 mg)	3	80	DMF	2.1 ± 0.5
KOH (5 mg)	3	80	DMF	4.5 ± 2.6
1 m NaOH (10 µL)	3	80	DMF/MDSO	10.2 ± 3.8
K_2_CO_3_ (5 mg)	3	60	DMF	41.8 ± 5.4
K_2_CO_3_ (5 mg)	3	80	DMF	22.1 ± 4.7

a) RCY was measured by HPLC (non‐decay corrected to trapped [^11^C]CH_3_I). Data are expressed as mean ± standard deviation (SD).

### 2.3 PET/CT Imaging of [^11^C]CNY‐10 in Wild‐Type Mice

**Figure 2:**
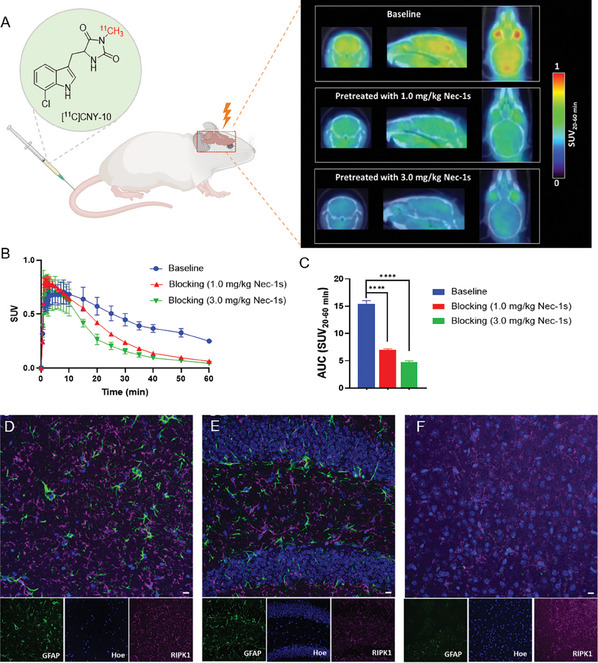
Detection and validation of RIPK1 analysis by PET/CT imaging and IHC. A) Representative mouse brain PET/CT images (summed 20‒60 min) were generated after intravenous administration (i.v.) of [^11^C]CNY‐10 in WT male mice of 3‐month‐old of age. B) Time‐activity curves of baseline and self‐blocking (pretreatment with unlabeled Nec‐1s 1.0 and 3.0 mg kg^−1^, i.v.). C) The area under the curve (AUC) of TACs in (B) shows the significantly decreased radioactivity accumulation in the brain of blocking mice. D–F) Immunofluorescence microscopy‐based IHC staining images of RIPK1 in the hippocampal *cornu ammonis* (D), dentate gyrus (E), and cortex (F).

Next, we conducted PET/CT imaging studies in wild‐type (WT) mice to evaluate the in vivo performance of [^11^C]CNY‐10. Previous studies indicated that different metabolism properties of Nec‐1s were noted in gender‐different mice, with more rapid clearance in female mice. As such, we chose male mice for our study^[^ ^33^ ^]^ In the PET imaging studies, male mice (C57BL/6) were administered 3.7–5.6 Mbq [^11^C]CNY‐10 and followed a 60‐min dynamic PET scan. The representative summed brain PET/CT images (20–60 min, and displayed as coronal, axial, and sagittal levels) and time‐activity curve (TAC) are shown in **Figure** **2**. [^11^C]CNY‐10 exhibited good brain uptake with a maximum standard uptake value (SUV) of 0.8. The TAC indicated that [^11^C]CNY‐10 has a fast brain penetration in the first few minutes after injection and washes out gradually, suggesting its suitable kinetics. To evaluate the binding specificity of [^11^C]CNY‐10, blocking studies were carried out. Mice were pretreated with unlabeled Nec‐1s in low (1.0 mg kg^−1^) or high (3.0 mg kg^−1^) doses via lateral tail vein 5 min before [^11^C]CNY‐10 injection. As the images and TACs showed in Figure 2, pretreatment with Nec‐1s resulted in significantly decreased brain uptake of [^11^C]CNY‐10 in a dose‐dependent manner, indicating high specific binding. Our first reported RIPK1 PET probe [^18^F]CNY‐07 exhibited good brain permeability and specificity^[^ ^30^ ^]^ Compared with [^18^F]CNY‐07, [^11^C]CNY‐10 showed increased brain uptake (SUV_max_: 0.8 vs 0.6) and demonstrated more favorable binding specificity and brain kinetics profiles (**Table** **3**). These findings give prominence to the potential for [^11^C]CNY‐10 to warrant additional translational exploration and assessment in the context of disease pathology.

**Table 3:** The comparison between [^11^C]CNY‐10 with [^18^F]CNY‐07.

Radioligand	Binding affinity to RIPK1 (K_d_)	Radiochemical yield (RCY)	In vivo binding specificity	Brain kinetic properties
[^18^F]CNY‐07	68 nm	23.5–28.5%	Moderate	Moderate
[^11^C]CNY‐10	3.1 nm	33.4–44.2%	Good	Good

We further performed immunofluorescence microscopy‐based immunohistochemistry (IHC) staining of mouse brain tissue to analyze RIPK expression. As a result, endogenous expression of RIPK1 in various brain areas, including hippocampal *cornu ammonis* (CA) (Figure 2D) and dentate gyrus (DG) (Figure 2E) as well as the cortex (Figure 2F) in the brain of male WT mouse of 3‐month‐old of age. While astrocytes were differentially expressed in the hippocampus and cortex, the RIPK1 signal was comparable across different brain areas and displayed a more uniform pattern than the GFAP signal by IHC. Since RIPK1 is a microglia‐dominant protein, this suggests a conserved function of microglia in the brain and also indicates that our new probe is more specific to microglia, other than astrocytes.

### 2.4 Non‐Human Primate (NHP) Imaging

**Figure 3:**
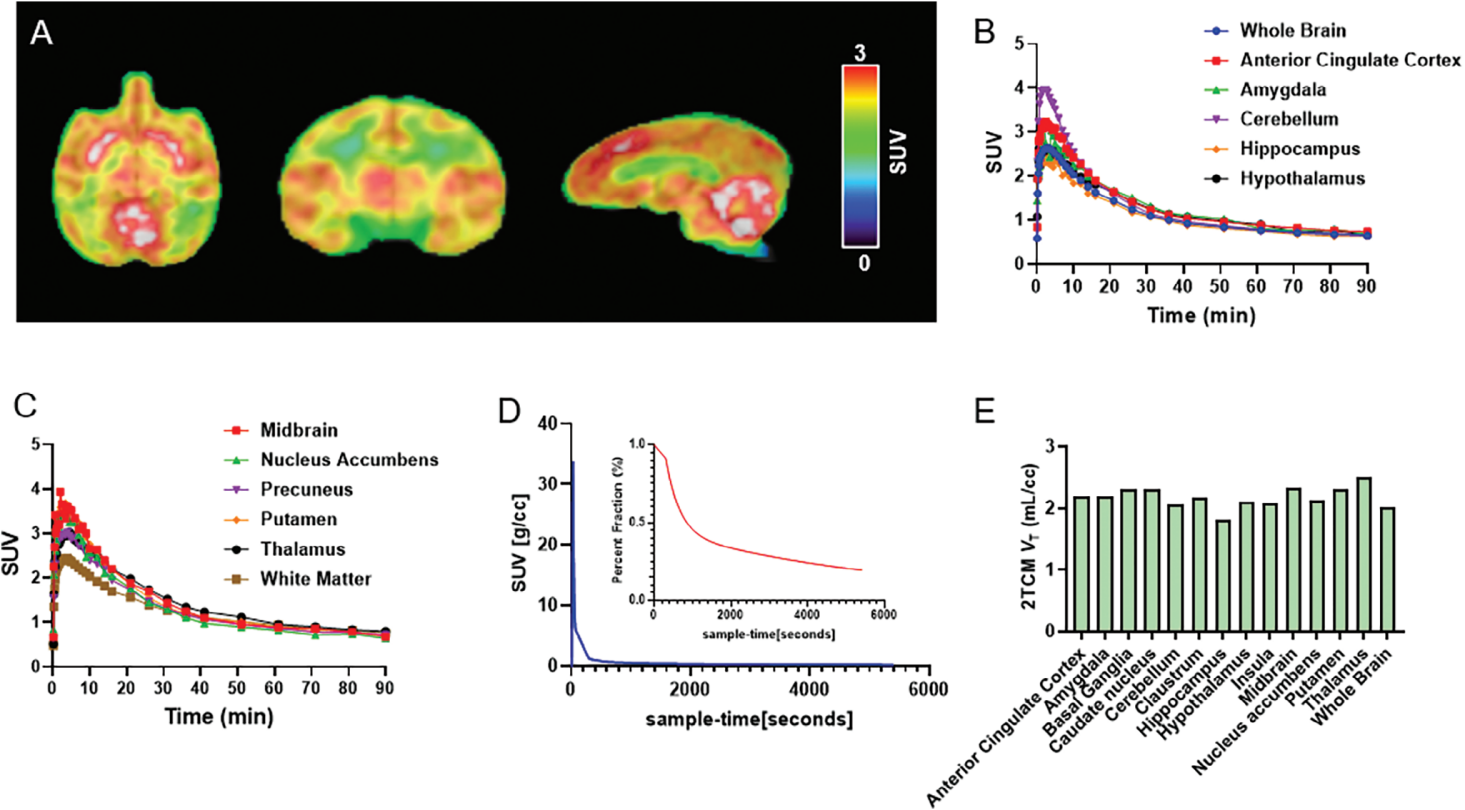
PET/MR imaging of [^11^C]CNY‐10 in NHP. A) Representative macaque brain PET/MR images of [^11^C]CNY‐10 (2 mm smoothing, averaged over 90 min). B,C) TACs of [^11^C]CNY‐10 in the whole brain and different brain regions; D) Arterial plasma analysis of [^11^C]CNY‐10; E) Two‐tissue compartmental model (2TCM) *V*_T_ values of regions of interest.

To assess the translational potential of [^11^C]CNY‐10 for the clinical practice of human brain RIPK1 imaging, we carried out PET imaging studies in a non‐human primate (NHP). In the NHP study, 90 min dynamic PET/magnetic resonance (MR) imaging acquisition was initiated after administration of [^11^C]CNY‐10 (4.33 mCi). Concomitantly, arterial blood was sampled at different time points for metabolism and kinetic analysis of [^11^C]CNY‐10. As shown in **Figure** **3**, high brain uptake of [^11^C]CNY‐10 in the NHP was observed with a whole brain peak SUV value of 2.6 post‐injection. TAC (Figure 3B) shows [^11^C]CNY‐10 penetrated the brain rapidly in the first few minutes after injection and washed out gradually, exhibiting similar kinetics profiles to mice. Regional analysis revealed a heterogeneous distribution of [^11^C]CNY‐10 across brain regions. Relatively high uptake was found in brain regions such as the cerebellum, putamen, midbrain, anterior cingulate cortex, and amygdala (Figure 3B,C).

Understanding the metabolism of [^11^C]CNY‐10 is very important for modeling its distribution kinetics and assessing safety profiles. Metabolite‐corrected plasma data were generated for kinetic modeling and distribution volume (*V*_T_) analysis. As shown in Figure 3D, 50% of the parent fraction of [^11^C]CNY‐10 was retained in plasma at 14 min and less than 35% at 30 min, indicating the rapid washout profile of [^11^C]CNY‐10 in blood. One‐ and Two‐tissue compartmental models (1TCM and 2TCM) were established for distribution volumes (*V*_T_) prediction. Akaike Information Criterion (AIC) and Model Selection Criterion (MSC) metrics suggested poor performance of 1TCM in quantifying [^11^C]CNY‐10 compared to 2TCM after Logan Plots (Table S1, Supporting Information). The *V*_T_ (2TCM) values of the 12 ROIs are shown in Figure 3E. The highest *V*_T_ was found in the thalamus at 2.52 mL cm^−3^, whereas the lowest was in the hippocampus at 1.81 mL cm^−3^. Future work will seek to extend the scope of this preliminary analysis by increasing the number of animals to achieve higher power statistical comparisons between kinetic models in baseline primate studies. Finally, multiple blocking studies and dynamic blood measurements with [^11^C]CNY‐10 in NHP will be performed to ensure the application of this tracer in human studies.

### 2.5 PET/CT Imaging of [^11^C]CNY‐10 in AD Mice

**Figure 4:**
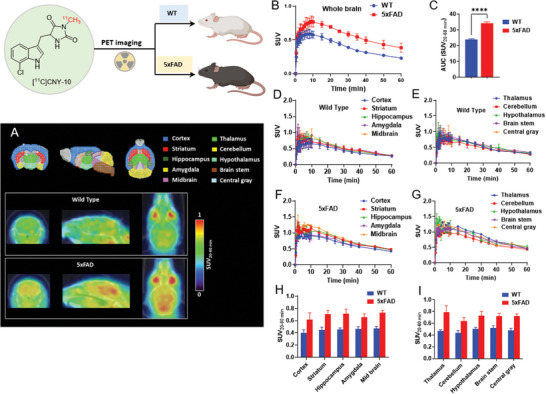
PET imaging studies of [^11^C]CNY‐10 in WT and 5xFAD mice. A) Representative PET images (summed 20–60 min) focused on the WT and 5xFAD mice brain. The regions of interest are shown as different colors in the mouse (Ma‐Benveniste‐Mirrione) VOI atlas template in the PMOD. B) TACs of [^11^C]CNY‐10 in WT and 5xFAD mice brain. C) The AUC of TACs in (B) shows the significantly increased radioactivity accumulation in the brain of AD mice. D,E) The TAC of [^11^C]CNY‐10 in ROIs of WT mice brain. F,G) The TAC of [^11^C]CNY‐10 in ROIs of 5xFAD mice brain. H,I) The biodistribution of [^11^C]CNY‐10 in ROIs of WT and 5xFAD mice brain. Results were quantified using Student's *t*‐test (^****^ *p* < 0.0001). Results were shown as mean ± SEM (*n* = 3).

The promising in vivo performance of [^11^C]CNY‐10 exhibited in rodents and NHP prompted us to conduct PET imaging studies in animal models to explore its diagnostic and target investigational potential. Given that RIPK1 has been found upregulated in AD, noninvasive PET imaging of RIPK1 in the brain may enable early evaluation of AD and advance the potential of drug development for AD and potentially other neurodegenerative diseases that display neuroinflammation associated with RIPK1 changes. Accordingly, we performed the PET imaging studies with [^11^C]CNY‐10 in the well‐established 5xFAD (APP^Swedish/Florida/London^, PSEN1^M146L/L286V^) transgenic mice (male, 8‐month‐old) and corresponding WT mice.^[^ ^34^, ^35^ ^]^ As a result, the brain uptake of [^11^C]CNY‐10 in AD mice was significantly higher than age and gender‐matched WT mice (**Figure** **4**A–C), consistent with the previous in vitro studies.^[^ ^22^, ^32^ ^]^ In addition, we investigated the regional biodistribution of [^11^C]CNY‐10 in mice brain using the mouse (Ma‐Benveniste‐Mirrione) VOI atlas in PMOD (an imaging analysis software).^[^ ^36^, ^37^ ^]^ Heterogeneous distribution of [^11^C]CNY‐10 was found in eight major functional regions: cortex, striatum, hippocampus, amygdala, midbrain, thalamus, cerebellum, hypothalamus, brain stem, and central gray in both WT and 5xFAD mice (Figure 4D–G). Among these regions of interest (ROIs), high radioactivity accumulation was observed in the striatum, hippocampus, and thalamus, whereas relatively low radioactivity was found in the cerebellum and hypothalamus. Compared with WT mice, the uptake of [^11^C]CNY‐10 increased across all ROIs in the 5xFAD mice brain, especially in the regions of the striatum, hippocampus, and thalamus, which are correlated with learning, memory, and cognition (Figure 4H,I). The PET imaging investigations utilizing [^11^C]CNY‐10 in WT and AD transgenic mouse models have unveiled the promising potential of our novel radioligand in characterizing AD and unraveled the intricacies of RIPK1‐related pathophysiology. Moreover, in stark contrast to the radioligand targeting the mitochondrial translocator protein (TSPO) known to primarily interact with glial cells,^[^ ^38^ ^]^ [^11^C]CNY‐10 exhibits remarkable specificity for RIPK1, a protein predominantly expressed in microglia and intricately linked to AD pathology. This distinctive feature not only sets [^11^C]CNY‐10 apart but also broadens the horizons of neuroinflammation detection in the context of AD, offering a novel avenue for exploring and understanding the underlying mechanisms of this debilitating disease.

### 2.6 Autoradiography Studies of [^11^C]CNY‐10

**Figure 5:**
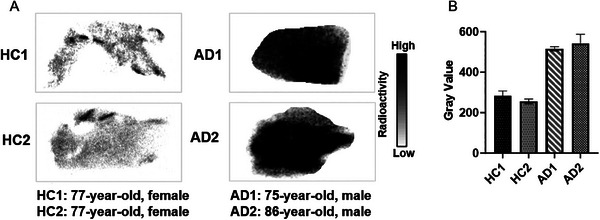
Autoradiography studies of [^11^C]CNY‐10. A) Autoradiographic images of [^11^C]CNY‐10 on human brain sections. B) The gray value of autoradiographic images indicates significantly increased radioactivity uptake of [^11^C]CNY‐10 in AD brain sections.

In addition to our studies using NHP models and AD mice, we further assessed the translational potential of our new probe [^11^C]CNY‐10 and carried out autoradiography studies in human postmortem brain sections for AD. We reasoned that results may further extend the potential clinical promise of [^11^C]CNY‐10 in AD. As shown in **Figure** **5**, the autoradiographic images clearly showed that AD patient brain sections displayed a higher radioactivity uptake related to health control (HC). Previous results showed a significant increase of RIPK1 expression in the brain of AD patients compared to the control.^[^ ^22^ ^]^ Collectively, our autoradiography results were consistent with previous findings, which further supported the translational potential of [^11^C]CNY‐10 for a clinical trial.

### 2.7 Ex Vivo Studies of RIPK1 Expression Changes in AD Transgenic Anismals

**Figure 6:**
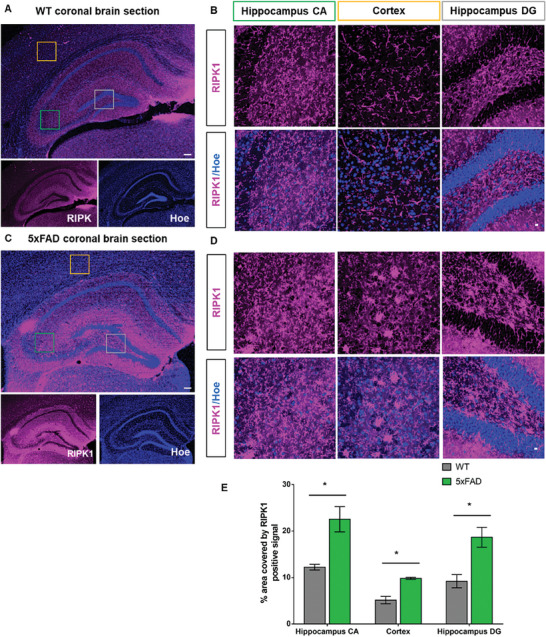
Immunohistochemical analysis of RIPK1 by immunofluorescence microscopy in AD transgenic animals compared to WT animals. AD‐related RIPK1 changes were analyzed by IHC staining using the RIPK1‐specific antibody in coronal brain sections of male WT and 5xFAD mice of 8‐month of age. Images were taken in a 4X confocal objective (Nikon C2), which demonstrated RIPK1 (in magenta) and nuclei by Hoechst (in blue) in separate and merged images. IHC analysis of RIPK1 was performed in WT animals A,B) and 5xFAD animals C,D), E) followed by quantification. The specific area within the sections was imaged under 4X objectives (A, C; scale bars = 100 µm) and then under 40X objectives at higher magnification (B, D; scale bars = 100 µm). Quantification of results was performed using Student's *t*‐test. Results were shown as the mean ± SEM.; *n* = 3; ^*^ *p* < 0.05.

Next, in parallel to the characterization of our new RIPK1 probe by PET for AD, we performed IHC assessments of RIPK1, focusing on AD‐related changes. Particularly, we carried out IHC on brain sections of both WT and 5xFAD of 8‐month‐old of age using RIPK1 as the primary antibody. We showed the expression of RIPK1 in different AD‐related brain regions, including the hippocampal CA and hippocampal DG and the cortex (**Figure** **6**A–D). RIPK1 expression was found to distribute throughout the brain sections of both the WT and 5xFAD transgenic (Tg) mice. The quantification of the microscopic images was performed, which revealed that RIPK1 expression was significantly increased across different brain areas examined in Tg mice compared to WT mice(Figure 6E). Our IHC results agreed with the previously reported increase of RIPK1 levels by biochemical analysis in AD mouse brains and were consistent with our PET imaging findings for RIPK1 expression.

Furthermore, because robust amyloid neuropathology usually manifests in 5xFAD mice starting 4–5 months of age in association with neuroinflammation,^[^ ^39^ ^]^ we investigated whether RIPK1 may change in the early stage of AD. This may help explore the potential of RIPK1 as a biomarker for early diagnosis and therapy evaluation of ADs. We analyzed 3‐month‐old female WT and 5xFAD mice and imaged different brain regions using a 40x confocal objective. Interestingly, we found significantly increased RIPK1 signal in various brain sub‐regions, including the hippocampus and cortex, compared to WT animals (Figure S2, Supporting Information). These data suggest that RIPK1 is increased in the early stage of AD and warrant future investigations using RIPK1 probes throughout the progression of AD in preclinical and potentially clinical settings.

**Figure 7:**
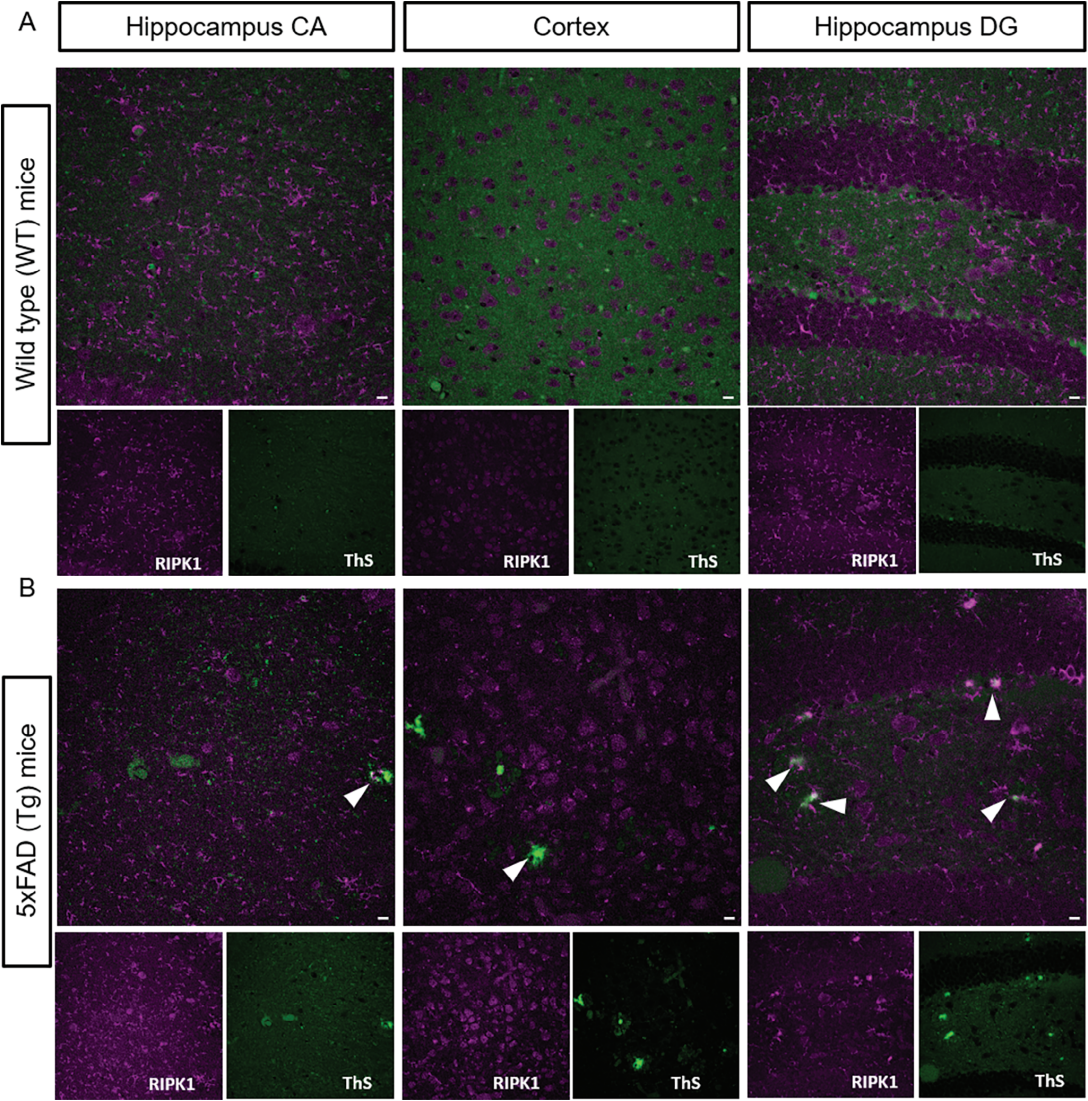
Immunohistochemistry (IHC) staining of RIPK1 in association with amyloid deposition. The analysis was performed using the RIPK1 specific antibody for RIPK1 (magenta) and β‐sheet amyloid specific thioflavin‐S (ThS) on coronal brain sections of wild‐type A) and 5xFAD transgenic mice B) of 8‐month‐old of age to observe the interaction of amyloid deposition with RIPK1. Images were captured in a 40X confocal objective (Nikon C2). Scale bars correspond to 10 µm. White arrow indicates the RIPK1 expression around amyloid deposition at different sub‐regions of brain sections.

Although both Aβ and tau pathology have been extensively investigated as essential biomarkers of AD, new biomarkers are required to be identified that can further help understand and intervene in the disease. Toward this end, our current study has indicated RIPK1 as a pathological biomarker for AD, focusing on characterizing and developing a new PET probe for RIPK1 imaging. Previous studies suggested that down‐regulation of RIPK1, by both pharmacological and genetic means, reduced amyloid pathology, corrected altered inflammatory changes, and attenuated memory deficits in the APP/PS1 transgenic mouse model and promoted microglial degradation of Aβ in a cell model.^[^ ^22^, ^40^ ^]^ To further explore the association of RIPK1 with amyloid plaques, we performed IHC analysis by probing RIPK1 in combination with thioflavin‐S (ThS), a molecule that has been commonly used to specifically probe the secondary β‐sheet structure of amyloid aggregation. We showed that ThS‐stained amyloid deposition was evidently shown in different brain regions of Tg mice, but not WT animals (**Figure** **7**A,B). Interestingly, RIPK1 closely engaged in interactions with ThS‐stained amyloid plaques in the brain, with the overlapping signal denoted by white arrows (Figure 7B). Moreover, we characterized the association of RIPK1 with amyloid plaques in AD animals through validation using Z‐scan analysis of IHC brain sections. We showed an orthogonal representation, which indicated the overlapping area of RIPK1 with ThS‐stained amyloid plaques within the brain sections (denoted with yellow‐headed arrows) (Figure S3, Supporting Information). Collectively, our ex vivo data are consistent with our PET imaging results and agree with previous findings, supporting a significant mechanism by which RIPK1 associates with amyloid plaque deposition in AD^[^ ^22^ ^]^

## 3 Conclusion

In this study, we developed a new PET radioligand [^11^C]CNY‐10 based on the potent RIPK1 inhibitor Nec‐1s for brain RIPK1 imaging. [^11^C]CNY‐10 was successfully radiosynthesized by reacting the dimethyl Nec‐1s precursor with [^11^C]methyl iodide. The radiochemical yield of [^11^C]CNY‐10 was remarkably improved after condition optimization. PET/CT imaging with [^11^C]CNY‐10 in wild‐type mice showed a high brain uptake and good binding specificity in self‐blocking studies. Notably, [^11^C]CNY‐10 exhibited a more suitable clearance kinetic profile than the previously reported RIPK1 tracer [^18^F]CNY‐07 in the mice PET imaging studies. Furthermore, [^11^C]CNY‐10 was investigated using a transgenic AD model to test its ability to detect disease‐related changes. Particularly, our study was further applied in the 5xFAD transgenic mice of AD for PET imaging. As a result, heterogeneous distribution and significantly higher uptake of [^11^C]CNY‐10 were observed in AD mice brain relative to wild‐type control mice, indicating the overexpression of RIPK1 in AD, consistent with previously reported in vitro results. Moreover, [^11^C]CNY‐10 showed high brain penetration and good kinetic modeling profiles in the brain of NHP, suggesting the translational potential of [^11^C]CNY‐10. Collectively, our results demonstrated [^11^C]CNY‐10 could be used as an important and valuable tool for investigating necroptosis and neuroinflammation in AD and potentially other CNS‐related diseases.

## 4 Experimental Section

All commercially available chemical reagents and solvents were of ACS–grade purity or higher and directly used without further purification. Nec‐1s and the precursor **1** were purchased from MedChemExpress. Anhydrous dimethyl formamide (DMF) was purchased from Acros Organics. Donkey serum (Sigma, #D9663‐10ML), DPBS (Gibco, 14190‐144), Triton‐X‐100 (Merck, #TX1568‐1), ProLong Gold antifade reagent (Invitrogen, # P36934) and RIPK1 primary antibody (1:100 dilution, Biotechne MAB3585); Hoechst (1:10 000 dilutions, Thermo Scientific), Donkey Alexa Fluor 640 tagged anti‐mouse secondary antibody (1:500 dilution, Jackson ImmunoResearch Lab. Inc.) and Thioflavin‐S (ThS) (0.1% dilution in PBS). Analytical separation was conducted on an Agilent 1100 series HPLC fitted with a diode‒array detector, quaternary pump, vacuum degasser, and autosampler.

[^11^C]CO_2_ (1.2 Ci) was obtained via the ^14^N (*p, α*) ^11^C reaction on nitrogen with 2.5% oxygen, with 11 MeV protons (Siemens Eclipse cyclotron, Siemens Healthcare GmbH, Erlangen, Germany), and trapped on molecular sieves in a TRACERlab FX‐MeI synthesizer (General Electric, G.E. Healthcare, Boston, MA, USA). [^11^C]CH_4_ was obtained by the reduction of [^11^C]CO_2_ in the presence of Ni/hydrogen at 350 °C and recirculated through an oven containing I_2_ to produce [^11^C]CH_3_I via a radical reaction.

All animal studies were carried out at Massachusetts General Hospital (PHS Assurance of Compliance No. A3596–01). The Subcommittee on Research Animal Care (SRAC) serves as the Institutional Animal Care and Use Committee (IACUC) for the Massachusetts General Hospital (MGH). SRAC reviewed and approved all procedures detailed in this paper.

Micro PET/CT imaging was performed in two groups: male C57BL/6 mice, used for general brain imaging and blocking studies; male 5xFAD transgenic mice and its age and gender‐matched WT mice, used as AD model. All mice were anesthetized by using isoflurane to minimize discomfort. Highly trained animal technicians monitored animal safety throughout all procedures, and the veterinary staff was responsible for daily care. All mice were socially housed in cages appropriate for the physical and behavioral health of the individual animal and were given unlimited access to food and water, with additional nutritional supplements provided as prescribed by the attending veterinary staff.

PET/MR imaging studies were performed with a male rhesus macaque (9.84 kg) that was deprived of food prior to the study and anesthetized by using intramuscular xylazine and ketamine to minimize discomfort. During imaging, the NHP was maintained with 1–2% isoflurane. Highly trained animal technicians monitored animal safety throughout all procedures, and veterinary staff were responsible for daily care. The rhesus macaque was socially housed in cage appropriate for the physical and behavioral health of the individual animal and was given unlimited access to food and water, with additional nutritional supplements provided as prescribed by the attending veterinary staff.

### Radiosynthesis of [^11^C]CNY‐10

[^11^C]methyl iodide ([^11^C]CH_3_I) was trapped in a TRACERlab FX‐M synthesizer reactor (General Electric) preloaded with a solution of precursor **1** in anhydrous DMF (2.0 mg mL^−1^, 0.3 mL) and K_2_CO_3_ (5 mg). The mixture was stirred at 60 °C for 3 min and then cooled down at room temperature within 1–2 min The reaction mixture was diluted with 0.1% trifluoroacetic acid (TFA) in water (1.2 mL) purified by reverse phase semipreparative HPLC (Agilent Eclipse XDB‐C18, 5 µm, 250 mm × 9.4 mm, flow rate = 5.0 mL min^−1^, mobile phase = 0.1% TFA in water/0.1% TFA in acetonitrile, 71/29, v/v), and the desired fraction was collected. The final product was reformulated by loading onto a solid‐phase exchange (SPE) C‐18 cartridge, rinsing with H_2_O (5 mL), eluting with ethanol (1 mL), and diluting with saline solution (0.9%, 9 mL). The average time required for the synthesis from the end of cyclotron bombardment to the end of synthesis was ≈40–50 min The radiochemical yield was 33.4% to 44.2% (nondecay corrected to trapped [^11^C]CH_3_I). Chemical and radiochemical purities were ≥95% with a specific activity of 305 GBq µmol^−1^ (E.O.B.).

### Mice PET/CT Acquisition and Post‐Processing

The PET/CT imaging studies in mice were referred to the previous work.^[^ ^41^, ^42^ ^]^ Briefly, Mice (WT: C57/BL6, male,8‐month‐old; AD: 5xFAD, male, 8‐month‐old, n = 6 for each group) were anesthetized with inhalational isoflurane (Patterson Vet Supply, Inc., Greeley, CO, USA) at 2% in a carrier of 2 L min^−1^ medical oxygen and maintained at 1% isoflurane for the duration of PET imaging scans. The mice were arranged side‐by‐side in two layers in a Triumph Trimodality PET/CT/SPECT scanner (Gamma Medica, Northridge, CA, USA). Mice were injected with [^11^C]CNY‐10 (3.7–5.6 Mbq) via lateral tail vein catheterization at the start of PET acquisition. For the blocking studies, mice were pretreated with Nec‐1s at the dose of 3.0 or 1.0 mg kg^−1^ via lateral tail vein 5 min before [^11^C]CNY‐10 injection. Dynamic PET acquisition lasted for 60 min, followed by computed tomography (CT) for anatomic co‐registration. PET data were reconstructed using a 3D‐MLEM method, resulting in full width at a half‐maximum resolution of 1 mm. Reconstructed images were exported from the scanner in DICOM format, along with an anatomic CT for rodent studies. These files were imported and analyzed using AMIDE (a medical imaging data examiner) software (an open‐source software, Los Angeles, CA, USA)^[^ ^43^ ^]^ and PMOD (PMOD 4.01, PMOD Technologies Ltd., Zurich, Switzerland).

### Mice PET/CT Image Analysis

Volumes of interest (VOIs) were generated manually in the form of spheres under the guide of high‐resolution C.T. structural images and summed PET data in mice brain regions, with a radius of no <1 mm, to minimize partial volume effects. Time‐activity curves (TACs) were exported as decay‐corrected activity per unit volume.

### NHP PET/MR Acquisition and Post‐Processing

The PET/MR imaging studies in NHP were referred to the previous work.^[^ ^44^, ^45^ ^]^ A male rhesus macaque was deprived of food for 12 h prior to the imaging study. Anesthesia was induced with intramuscular xylazine (0.5–2.0 mg kg^−1^) and ketamine (10 mg kg^−1^). After endotracheal intubation, V‐line and A‐line were inserted and an anesthetic state was maintained using isoflurane. The macaque was antecubital catheterized for radiotracer injection, and a radial arterial line was placed for metabolite analysis. PET/MR images of the brain were acquired in a 3T Siemens TIM‐Trio with a BrainPET insert (Siemens, Munich, Germany) with a custom‐built 8‐channel head coil, isotropic PET resolution of 1.25 mm. While in the imaging system, the NHP was maintained at 1–2% isoflurane. Dynamic PET image acquisition was initiated, followed by the administration of [^11^C]CNY‐10. Before scanning, 4.33 mCi of [^11^C]CNY‐10 was administered to the macaque. An MEMPRAGE sequence was acquired near the end of PET imaging for anatomic co‐registration. Dynamic data from the PET scans were recorded in list mode and corrected for attenuation. Macaque data were reconstructed using a 3D‐OSEM method.

### NHP PET/MR Image Analysis

PET data were motion‐corrected, spatially smoothed with a 2.0 mm FWHM Gaussian filter, and registered to the INIA19 Template and NeuroMaps Atlas for brain imaging analysis.^[^ ^46^ ^]^ Image registration was carried out on high‐resolution MEMPRAGE MRI images using a twelve‐degree‐of‐freedom linear algorithm and a nonlinear algorithm to the atlas brain. The transformation was then applied to the dynamic PET data that had been collected simultaneously. Kinetic modeling was carried out in PMOD (PMOD 3.9, PMOD Technologies Ltd., Zurich, Switzerland). VOIs were selected according to the macaque brain atlas. A common VOI mask was applied to both baboon scans. TACs were exported from the whole brain, cerebellum, primary motor cortex, putamen, thalamus, primary visual cortex, caudate, and white matter VOIs for analysis. Logan Plots, 1TCM, and 2TCM models were used to estimate the regional volume of distribution (*V_T_*) with a metabolite‐corrected plasma TAC and assessed with goodness of fit analysis. Representative voxel‐wise *V_T_* maps were calculated using Logan plot results from the dynamic PET data.

### Plasma and Metabolite Analysis

Arterial blood samples were collected while the macaque underwent PET imaging. Metabolite extraction and High‐Performance Liquid Chromatography (HPLC) were based on previously reported methods from the institution to generate radioHPLC chromatograms for each blood sample.^[^ ^44^, ^45^ ^]^ Briefly, blood samples were centrifuged to isolate plasma. Protein precipitation was attained via the addition and mixing of plasma (1 mL) to acetonitrile (1 mL). This was then centrifuged for 1 min to obtain protein‐free plasma. The protein‐free supernatant (1 mL) was diluted in deionized water (4 mL) and underwent HPLC to distinguish radiometabolites from the parent radiotracer. HPLC setup included a column‐switching valve for sample concentration (online solid‐phase extraction; Agilent Bond Elut Online SPE, PLRP‐S, 4.6 × 12.5 mm) and subsequent separation (Agilent Eclipse Plus C18, 4.6 × 100 mm. 3.5 µm). Radiometabolite HPLC was conducted as follows: Plasma samples were placed onto the SPE concentrator column, 1% ACN, and 99% H_2_O at 2 mL min^−1^. After 3 min, the sample flowed from the SPE column to the separation column under gradient conditions (Mobile Phase A: water + 0.1% formic acid; Mobile Phase B: acetonitrile + 0.1% formic acid; separation method = 95/5 – 55/45 A/B from 3 – 8 min linear gradient; 55/45 – 5/95 A/B from 8–10 min linear gradient; 5/95 A/B from 10 to 11 min isocratic; flow rate 2 mL min^−1^). Analytes were then monitored for ≈10 min after sample injection via dual opposing bismuth germanium oxide detectors for coincidence detection (Eckert and Ziegler). Once generated, chromatograms were corrected for radioactive decay and subsequently integrated to quantify the area under the curve for each respective metabolite and comparison to the original parent tracer. Each plasma sample's parent fraction was fit and then applied to the plasma input curve, allowing derivation of the radiometabolite‐corrected plasma input function used for tissue compartment modeling discussed previously.

### Autoradiography

The in vitro autoradiography assay has been described in the previous report.^[^ ^47^ ^]^ Briefly, the human brain sections (From MIND Tissue Bank (https://www.massgeneral.org/neurology/mind/scientists/tissue‐bank) HC1 and HC2: 77‐year‐old, female; AD1: 75‐year‐old, male; AD2: 86‐year‐old, male) were pre‐incubated with Tris‐HCl buffer (50 mm) solution for 20 min, followed by incubation with [^11^C]CNY‐10 (1 mCi L^−1^, 50 mm Tris‐HCl buffer). Following incubation, brain sections were washed in an ice‐cold buffer and then dipped in ice‐cold distilled water. The brain sections were dried at ambient temperature. An imaging plate (BAS‐MS2025, GE Healthcare, NJ, USA) was exposed to the dried brain sections. Autoradiograms were obtained, and the gray value of the images was measured using ImageJ software.

### Immunohistochemistry (IHC)

IHC was performed using previously reported methods.^[^ ^39^, ^44^, ^48^, ^49^ ^]^ Briefly, the wild type (WT) and 5xFAD (Tg) mouse brain sections (*n* = 3) were used, which were washed four times with PBS (≈7 min each), and then blocked by incubation in donkey‐blocking serum solution (5% donkey serum + 0.3% Triton X‐100 in PBS) for over an hour at room temperature (r.t.) in a shaker with gentle shaking to eliminate non‐specific staining. Next, the blocking buffer was carefully discarded, and the primary antibody solution of anti‐mouse RIPK1 (1:100 dilution in 2.5% donkey serum with 0.3% Triton X‐100 in PBS) was added and incubated overnight in 4 °C with gentle shaking. Subsequently, brain sections were washed with PBS 4 times (≈7 min each) and incubated in a secondary antibody mixture (1:500 dilution of Alexa‐640 labeled anti‐mouse IgG antibodies prepared in 2.5% donkey serum with 0.3% TritonX‐100 in PBS) at r.t. for 2 h with gentle shaking. Brain sections were then washed with PBS twice (≈10 min each) and then incubated in 2 nm Hoechst contained in PBS for 10 min. For ThS staining, brain sections were incubated in 0.1% ThS (prepared in PBS and filtered through 0.22 µm syringe filter) for 10 min followed by washing with PBS for over 10 times (≈5 min each), till the yellow color of the washed solution became clear. Finally, brain sections were carefully mounted on a glass slide with an Invitrogen Prolong Gold antifade reagent after completely draining PBS, covered with a coverslip, and imaged in a Nikon C2 confocal microscopy.

### Statistical Analysis

Confocal microscopic images were processed and analyzed using Image J software. All the results for different experiments in this work were shown as mean ± SEM. Statistical significances of different results were performed using two tailed Student's *t*‐test in GraphPad PRISM 10.1.2. Values were considered as significant when it comes *p* < 0.05.

## Conflict of Interest

The authors declare no conflict of interest.

## Supporting information

Supporting Information

## Data Availability

The data that support the findings of this study are available on request from the corresponding author. The data are not publicly available due to privacy or ethical restrictions.
