# Supplementary material for: Development of a New Positron Emission Tomography Imaging Radioligand Targeting RIPK1 in the Brain and Characterization in Alzheimer's Disease
Source: Adv Sci (Weinh). 2024 Jun 25;11(32):2309021. doi: 10.1002/advs.202309021 (PMC11348174; doi:10.1002/advs.202309021)

ADVANCED SCIENCE
Open Access

Supporting Information

for *Adv. Sci.*, DOI 10.1002/advs.202309021

Development of a New Positron Emission Tomography Imaging Radioligand Targeting RIPK1 in the Brain and Characterization in Alzheimer's Disease

Ping Bai, Yu Lan, Yan Liu, Prasenjit Mondal, Ashley Gomm, Yulong Xu, Yanli Wang, Yongle Wang, Leyi Kang, Lili Pan, Frederick A. Bagdasarian, Madelyn Hallisey, Fleur Lobo, Breanna Varela, Se Hoon Choi, Stephen N. Gomperts, Hsiao-Ying Wey, Shiqian Shen, Rudolph E. Tanzi, Changning Wang* and Can Zhang**

Supplementary Materials

Development of a new positron emission tomography imaging radioligand targeting RIPK1 in the brain and characterization for the use in Alzheimer's disease

Ping Bai[a,*], Yu Lan[b,c], Yan Liu[b], Prasenjit Mondal[d], Ashley Gomm[d], Yulong Xu[b], Yanli Wang[b], Yongle Wang[b], Frederick A. Bagdasarian[b], Madelyn Hallisey[b], Fleur Lobo[d], Breanna Varela[b], Se Hoon Choi[d], Stephen N. Gomperts[e], Hsiao-Ying Wey[b], Rudolph E. Tanzi[d], Changning Wang[b,*], Can Zhang[d,*]

[a]*Department of Pulmonary and Critical Care Medicine, Targeted Tracer Research and Development Laboratory, Institute of Respiratory Health, Frontiers Science Center for Disease-related Molecular Network, Precision Medicine Key Laboratory of Sichuan Province & Precision Medicine Research Center, West China Hospital, Sichuan University, Chengdu, 610041, Sichuan, China*

[b]*Athinoula A. Martinos Center for Biomedical Imaging, Department of Radiology, Massachusetts General Hospital, Harvard Medical School, Charlestown, Massachusetts 02129, United States*

[c]*Department of Pharmacy, Renmin Hospital of Wuhan University, Wuhan 430060, China*

[d]*Genetics and Aging Research Unit, McCance Center for Brain Health, MassGeneral Institute for Neurodegenerative Disease, Department of Neurology, Massachusetts General Hospital, Harvard Medical School, 114 16th Street, Charlestown, MA 02129, United States*

[e]*Department of Neurology, Massachusetts General Hospital, Harvard Medical School, 114 16th Street, Charlestown, MA 02129, United States*

Table of Contents

General procedure for preparation of compound 2 3

General procedure for preparation of precursor 3 3

Figure S1. The HPLC chromatogram of [^{11}C]CNY-10 and unlabeled CNY-10 4

Figure S2: Immunofluorescence microscopy-based IHC staining on brain sections of wild-type (WT) and 5xFAD mice 5

Figure S3: Confocal Z-stack analysis of RIPK1 in association with amyloid deposits by IHC on brain sections of 5xFAD animals 6

Table S1. Kinetic modeling of [^{11}C]CNY-10 7

NMR spectra for the synthesized compounds 8

General procedure for preparation of compound 2

To a solution of 77-chloro-1H-indole-3-carbaldehyde (1 g, 6.13 mmol, 1 *eq*) in piperidine (8 mL) was added imidazolidine-2,4-dione (1.22 g, 12.26 mmol, 2 *eq*). The mixture was stirred at 110 °C for 6 hr under N_2. After cooling to room temperature, the residue was diluted with H_2O (80 mL), filtered and the filter cake was concentrated under reduced pressure to give compound **2** as a yellow solid. (Z)-5-((7-chloro-1H-indol-3-yl)methylene)imidazolidine-2,4-dione (**2**). ^{1}H NMR (400 MHz, DMSO-d_6) δ 12.03 (s, 1H), 10.43 (s, 1H), 8.22 (s, 1H), 7.83 (d, J = 7.9 Hz, 1H), 7.41 (d, J = 7.5 Hz, 1H), 7.07 (t, J = 7.8 Hz, 1H), 6.81 (s, 1H). M.S. (ESI+): 263.2 $(M + H)^+$.

General procedure for preparation of precursor 3

To a solution of intermediate **2** (0.4 g, 1.53 mmol, 1 *eq*) in T.H.F. (10 mL) and H_2O (10 mL)was added $CoCl_2$ (400.68 mg, 3.09 mmol, 2 *eq*). After cooling down to 0 °C in ice water bath, the mixture was added $NaBH_4$ (875.63 mg, 23.14 mmol, 15 *eq*). The mixture was stirred at 20 °C for 12 hr under N_2. The reaction mixture was diluted with H_2O (30 mL) and extracted with D.C.M. (50 mL * 2). The combined organic layers were dried over Na_2SO_4, filtered, and the filtrate was concentrated under reduced pressure to give a residue. The residue was purified by prep-HPLC (column: Welch Ultimate AQ-C18 150*30mm*5um; mobile phase: [water (HCl)-A.C.N.]; B%:17%-47%,10min). Precursor **3** was obtained as a yellow solid. 5-((7-chloro-1H-indol-3-yl)methyl)imidazolidine-2,4-dione (**3**). ^{1}H NMR (400 MHz, DMSO-d_6) δ 11.14 (s, 1H), 8.18 (s, 1H), 7.55 (d, J = 7.9 Hz, 1H), 7.28 (d, J = 7.5 Hz, 1H), 7.18 (d, J = 2.3 Hz, 1H), 6.93 (t, J = 7.7 Hz, 1H), 4.36 (t, J = 4.8 Hz, 1H), 3.08 (qd, J = 14.8, 5.1 Hz, 2H). M.S. (ESI+): 265.1 $(M + H)^+$.

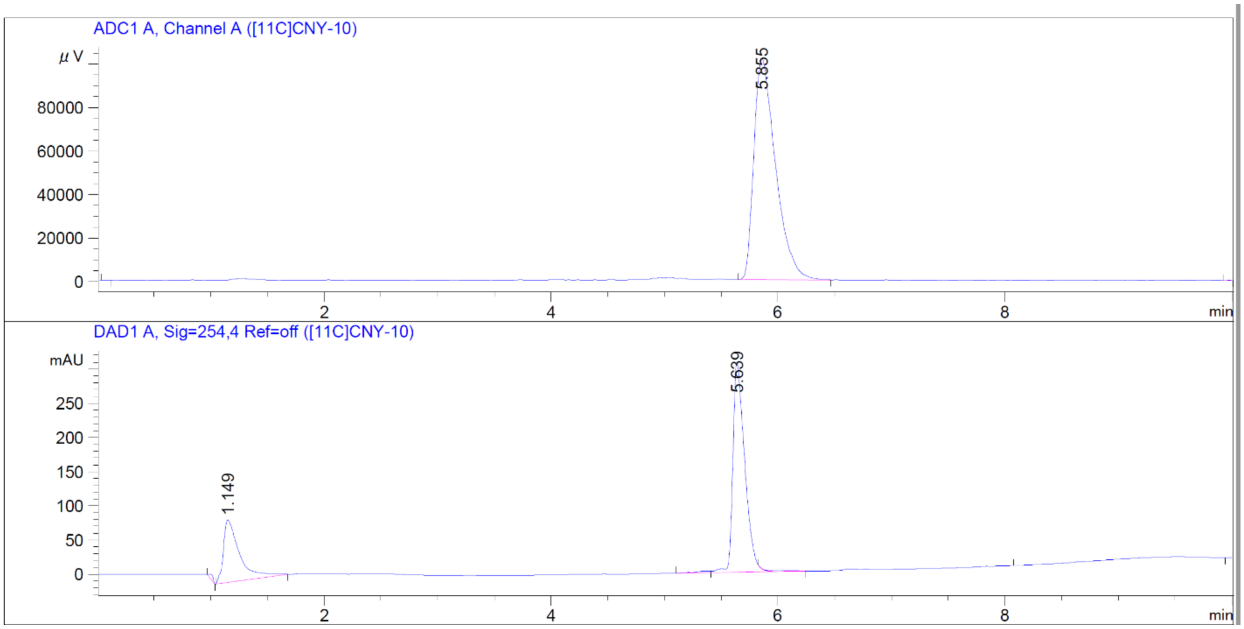

Figure S1. The HPLC chromatogram of [^{11}C]CNY-10 and unlabeled CNY-10. Analytic HPLC condition: Agilent Eclipse plus C18, 3.5 μm, 4.6×100 mm, flow rate = 1.0 mL/min, mobile phase = 0.1% formic acid in water / 0.1% formic acid in acetonitrile, gradient method.

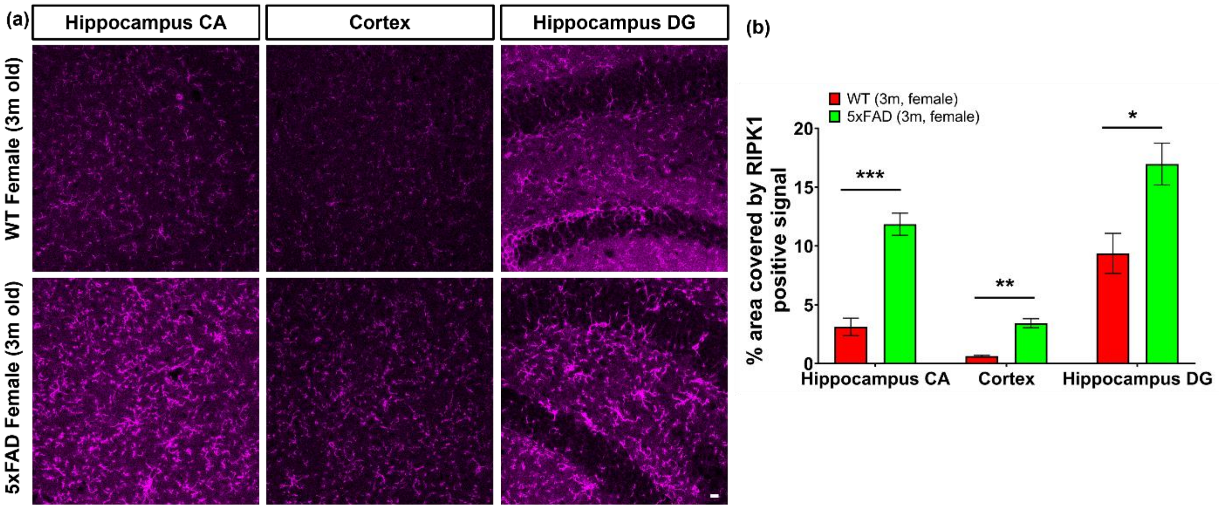

Figure S2: Immunofluorescence microscopy-based IHC staining of brain sections from 3-month-old of age wild-type (WT) and 5xFAD female mice using RIPK1 specific antibody for RIPK1 in magenta. (a) Images were taken from different brain regions using a 40X confocal objective (Nikon C2) and scale bar corresponded to 10 µm. (b) Quantifications of IHC images were performed in Image J and shown as mean ± SEM, n=3; asterisks showed statistical significances; *p<0.05, **<0.001, ***p<0.0001.

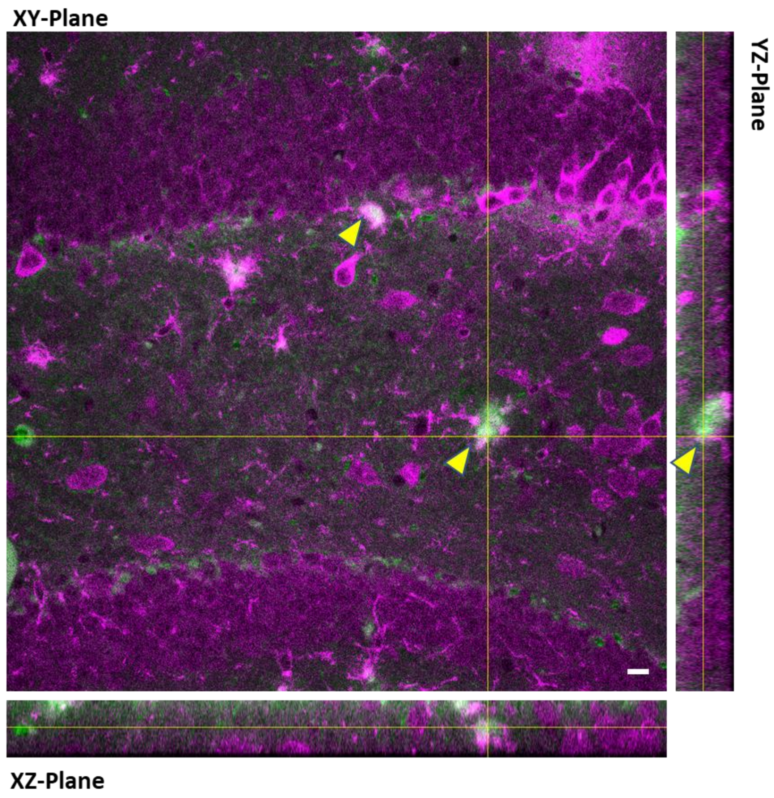

Figure S3: Confocal Z-stack analysis of RIPK1 in association with amyloid deposits by IHC in brain sections of 5xFAD animals. Orthogonal projections of confocal Z-scanned images shown as merged confocal stacks that captured at different laser channels. The yellow arrowhead represented the overlapping region of RIPK1 with amyloid deposits. Scale bar corresponded to 10 μm.

ROI	1TCM			2TCM		
VoiName(Region) [string]	AIC [1/1]	SC [1/1]	MSC [1/1]	AIC [1/1]	SC [1/1]	MSC [1/1]
ACC	53.19	55.78	2.57	29.01	33.57	3.33
Amygdala	59.26	61.86	2.01	49.91	54.46	2.33
Basal_Ganglia	58.23	60.82	2.41	33.43	37.99	3.19
Caudate	64.81	67.41	2.11	41.47	46.03	2.85
Cerebellum	69.94	72.53	2.41	14.44	19.00	4.13
Claustrum	54.68	57.28	2.42	32.39	36.94	3.13
Corpus_Callosum	66.88	69.47	1.46	34.46	39.02	2.48
Hippocampus	64.37	66.96	1.91	32.53	37.08	2.91
Hypothalamus	59.89	62.48	2.08	29.77	34.33	3.02
IFC	63.23	65.82	2.25	53.35	57.91	2.58
Insula	47.44	50.03	2.69	25.61	30.17	3.38
Midbrain	56.42	59.02	2.59	20.67	25.23	3.71
Motor	62.21	64.80	2.27	50.57	55.13	2.65
NAc	56.41	59.00	2.51	29.13	33.69	3.37
Occipital_Gyrus	77.62	80.21	2.34	26.95	31.51	3.90
OFC	62.85	65.45	2.21	47.40	51.95	2.71
PCC	62.71	65.30	2.08	49.20	53.76	2.52
Precuneus	59.82	62.41	2.31	46.95	51.51	2.73
Putamen	55.75	58.34	2.56	31.55	36.11	3.32
Sensory	62.44	65.04	2.32	51.17	55.72	2.70
Splenium	76.36	78.95	1.12	67.43	71.99	1.42
Thalamus	55.91	58.51	2.24	44.27	48.82	2.62
VTA	67.78	70.37	1.96	44.30	48.86	2.70
White_Matter	69.09	71.68	1.70	58.24	62.80	2.06
WB	65.89	68.48	2.12	43.18	47.74	2.84

Table S1. Kinetic modeling of [^{11}C]CNY-10. AIC: Akaike Information Criterion; SC: Schwartz Cirterion; MSC: Model Selection Criterion; Lower AIC, Lower SC, Higher MSC = better fits.

NMR spectra for the synthesized compounds:

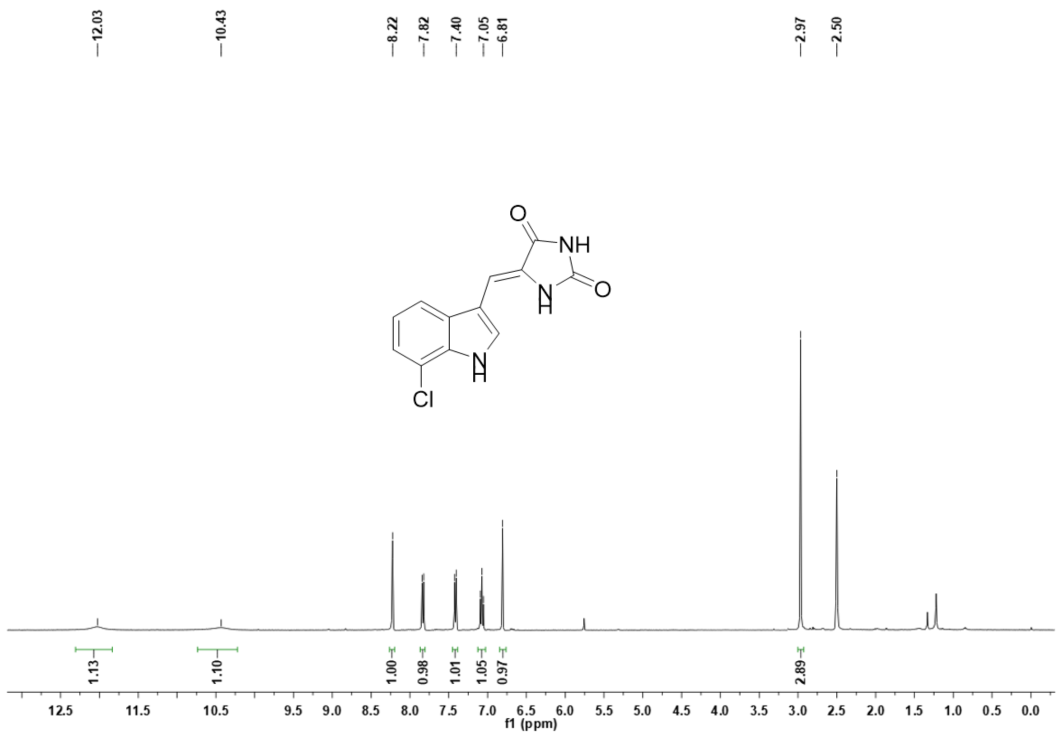

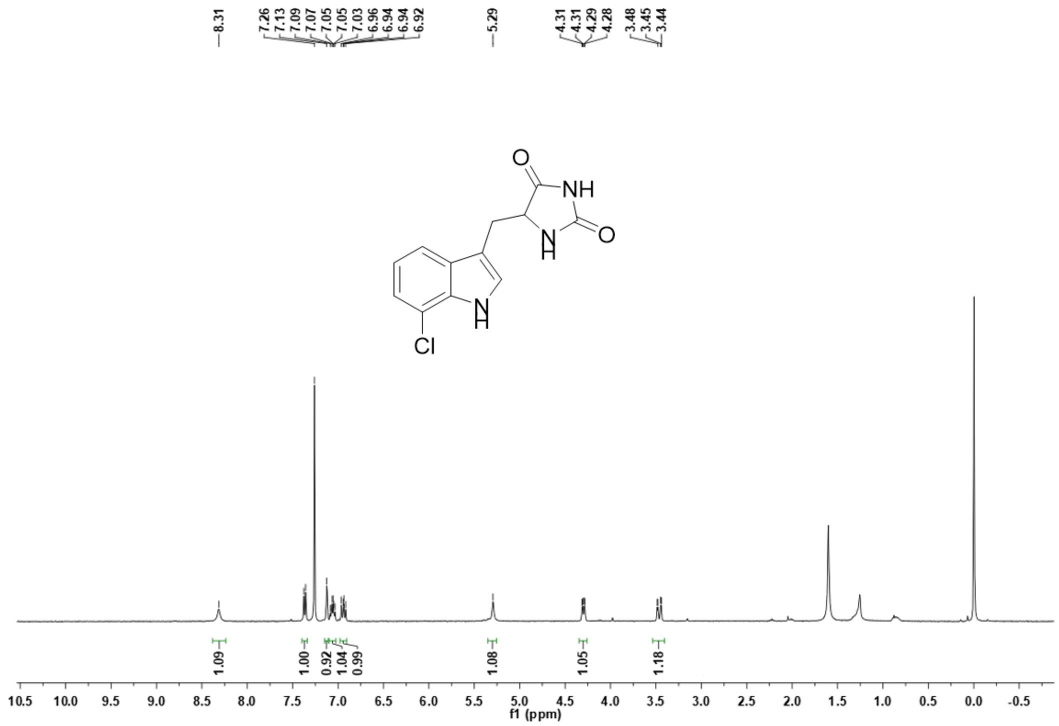

Supplement: Supplementary file 1 — Supporting Information [file ADVS-11-2309021-s001.pdf]
